# Comparing transcriptome expression profiles to reveal the mechanisms of salt tolerance and exogenous glycine betaine mitigation in maize seedlings

**DOI:** 10.1371/journal.pone.0233616

**Published:** 2020-05-29

**Authors:** Fenqi Chen, Peng Fang, Wenjing Zeng, Yongfu Ding, Zelong Zhuang, Yunling Peng

**Affiliations:** 1 College of Agronomy, Gansu Agricultural University, Lanzhou, China; 2 Gansu Provincial Key Lab of Aridland Crop Science, Lanzhou, China; Louisiana State University College of Agriculture, UNITED STATES

## Abstract

Salt stress is a common abiotic stress that limits the growth, development and yield of maize (*Zea mays* L.). To better understand the response of maize to salt stress and the mechanism by which exogenous glycine betaine (GB) alleviates the damaging effects of salt stress, the morphology, physiological and biochemical indexes, and root transcriptome expression profiles of seedlings of salt-sensitive inbred line P138 and salt-tolerant inbred line 8723 were compared under salt stress and GB-alleviated salt stress conditions. The results showed that under salt stress the growth of P138 was significantly inhibited and the vivo ion balance was disrupted, whereas 8723 could prevent salt injury by maintaining a high ratio of K^+^ to Na^+^. The addition of a suitable concentration of GB could effectively alleviate the damage caused by salt stress, and the mitigating effect on salt-sensitive inbred line P138 was more obvious than that on 8723. Transcriptome analysis revealed that 219 differentially expressed genes (DEGs) were up-regulated and 153 DEGs were down-regulated in both P138 and 8723 under NaCl treatment, and that 487 DEGs were up-regulated and 942 DEGs were down-regulated in both P138 and 8723 under salt plus exogenous GB treatment. In 8723 the response to salt stress is mainly achieved through stabilizing ion homeostasis, strong signal transduction activation, increasing reactive oxygen scavenging. GB alleviates salt stress in maize mainly by inducing gene expression changes to enhance the ion balance, secondary metabolic level, reactive oxygen scavenging mechanism, signal transduction activation. In addition, the transcription factors involved in the regulation of salt stress response and exogenous GB mitigation mainly belong to the MYB, MYB-related, AP2-EREBP, bHLH, and NAC families. We verified 10 selected up-regulated DEGs by quantitative real-time polymerase chain reaction (qRT-PCR), and the expression results were basically consistent with the transcriptome expression profiles. Our results from this study may provide the theoretical basis for determining maize salt tolerance mechanisms and the mechanism by which GB regulates salt tolerance.

## Introduction

Salt stress is one of the main abiotic stresses limiting the growth and development of plants. Soil salinization is an increasingly serious environmental and ecological problem that threatens the limited soil resources on which human beings depend for survival [1], and has a profound impact on the sustainability of crop yields [2]. Most plant species are sensitive to salt stress, which can cause ion toxicity, metabolic damage, osmotic stress and oxidative damage [3, 4]. The increase of salt concentration in plants will cause mitochondria, chloroplasts and other organelles to produce reactive oxygen species (ROS), which damage proteins, DNA, cell membranes and so on, and in turn affect the growth and development of plants [5]. However, plants have evolved a series of complex salt tolerance mechanisms involving complex physiological changes [6]. These mechanisms involve the expression and coordination of many genes, such as *SKC1*, a sodium transporter that participates in the response to salt stress by regulating the homeostasis of K^+^/Na^+^ [7], and *OsMAPK33*, a gene in the mitogen-activated protein kinase (MAPK) signal cascade that allows rice plants to cope with stress by maintaining cell osmotic balance [8]. A full understanding of the complex mechanisms by which plants respond to salt stress will help us to breeding plants that can better adapt to salt stress.

Maize (*Zea mays* L.) is a typical C4 crop with high yield and photosynthetic efficiency. It is one of the most important agricultural cash crops in the world because its raw materials are used to produce human and animal food as well as biofuels [9–11]. Unfortunately, it is not a salt-tolerant crop [12]. Salt stress leads to slow growth of maize seedlings, low survival rate, and damage to the photosynthetic system, and affects later development and yield [3, 13]. With the development of high-throughput sequencing technology, transcriptome profiling has been widely used to study the mechanism of plant responses to abiotic stress. Li *et al*. [14] analyzed the transcriptional expression profile of the roots of *Medicago truncatula* seedlings grown under 180 mM NaCl stress, and found that a new transcription factor (TF) gene *MtCBF4* played an important role in abiotic stress response. When this gene was overexpressed in *M*. *truncatula*, it successfully activated the expression of downstream genes and improved the tolerance of plants to salt stress. Peng *et al*. [15] analyzed the differential expression of genes in the leaves of salt-tolerant cotton (*Gossypium hirsutum* L.) and salt-sensitive cotton subjected to 24 hours of salt stress using a comparative transcriptome method; 819 TF genes were identified as differentially expressed in the two genotypes and 129 genes were identified as specifically expressed in the salt-tolerant genotype. Comparing the expression levels of starch metabolism genes in rice (*Oryza sativa* L.) seedlings of salt-tolerant and salt-sensitive genotypes under 200 mM NaCl stress, Theerawitaya *et al*. [16] found that the content of soluble sugar in the seedlings of both genotypes increased, and this increase was related to starch degradation. At the same time, they speculated that a major source of carbon mainly due to photosynthesis and starch metabolism in salt-tolerant genotypes. It can be seen that by comparing the physiological and molecular differences of two materials with significant salt tolerance differences under salt stress, the salt tolerance mechanism of plants can be effectively revealed. Therefore, comparing the differentially expressed genes (DEGs) in seedling roots of maize inbred lines with different salt tolerances would be an effective way to explore the mechanisms of salt tolerance.

Glycine betaine (GB) is a plant osmotic protective agent synthesized by choline oxidation or glycine methylation [17]. Under high salt conditions, GB can maintain osmotic pressure in cells, improve the ability of antioxidant damage, and affect the distribution of inorganic salt ions, enhancing the ability of both animals and plants to resist salt stress [18, 19]. For example, GB can improve the ability of halophilic bacteria strain PT-20 to degrade phenol. Under 10% NaCl, PT-20 completely degrades 1000 mg·L^-1^ phenol within 120 h. After adding GB, the time was shortened to 72 h, and the corresponding average degradation rate was increased from 8.43 to 14.28 mg·L^-1^·h^-1^. The results of transcriptome analysis showed that the addition of GB enhanced the resistance of cells to phenol; furthermore, GB increased the growth rate of strain PT-20 and up-regulated the expression of related enzyme genes [20]. Although researchers have conducted many studies on the effect of exogenous GB on plant responses to stress, there have been few transcriptome studies aimed at revealing the mechanism by which GB mitigates the effects of salt stress in maize. Therefore, we used transcriptome sequencing to compare and analyze the DEGs and TF families in two maize inbred lines (salt-tolerant 8723 and salt-sensitive P138) under salt stress and exogenous GB-alleviated salt stress conditions. The DEGs we identified included salt tolerance-related genes, such as potassium transporter 21-like, Ent-cassadiene C2-hydroxylase-like, and peroxidase 2, and members of TF families, such as MYB, MYB-related, AP2-EREBP, bHLH, NAC, and WRKY. The results from this study are helpful for clarifying the mechanism underlying salt tolerance in maize and the mechanism by which exogenous GB alleviates salt stress, and lay the foundation for the cultivation of new salt-tolerant maize varieties.

## Materials and methods

Salt-sensitive inbred line P138 and salt-tolerant inbred line 8723 were used as materials to study the mechanisms underlying salt tolerance and GB mitigation in maize. The selection of these two inbred lines and treatment methods was supported by the results of a previous study [21]. The experimental treatments were as follows: normal conditions (CK): 0 mM NaCl, salt stress treatment (S): 180 mM NaCl, GB mitigation treatment (T): 10 mM GB + 180 mM NaCl. The seeds were sterilized with 0.5% NaClO for 10 minutes and washed with distilled water five times. The seeds of the two inbred lines were soaked in different treatment solutions at 25°C for 12 h, then sowed in pots (15 cm × 13 cm, 10 plants per pot). There were three biological replicates for each treatment. After sowing, the seeds were cultured in a greenhouse at a temperature of 25 ± 2°C, 12-h photoperiod, light intensity of 600 μmol/s^−1^·m^−2^, and relative humidity of 60%, and deionized water (50 mL) was added every other day. In addition, 50 mL of treatment solution (CK, S, or T) was added every three days until the control leaves grew to the three-leaf stage. The roots of plants in each treatment group were washed with distilled water and then quickly placed in liquid nitrogen. The samples were stored at -80°C for further use.

### Index determination

#### Growth index

(1) Determination of plant height, root length and root number: plant height and the length of the main root of seedlings were measured with a straight ruler. (2) Determination of fresh weight and dry weight: maize seedlings were carefully removed from the pots, the vermiculite attached to the roots was rinsed off with water, the excess water on the surface was absorbed with filter paper, and the root system was separated from the aboveground parts with a double-sided razor blade. The aboveground and underground parts were then weighed on a balance. After weighing, the aboveground and underground parts were placed in the oven and dried at 105°C for 30 min, then 80°C to a constant weight, and the dry weight was measured. Five plants were sampled for all varieties and treatments for measurement.

#### Ion content determination

After about 10 days of stress treatment, when the control leaves reached the three-leaf stage, the maize seedlings from each treatment were carefully removed from the nutrition bowl, the vermiculite attached to their roots was washed with water, and the roots, stems and leaves were separated with a double-sided blade. The tissue was placed in a 105°C oven for 30 min, and then baked at 80°C for 48 h. The contents of Na^+^, K^+^ and Ca^2+^ ions in roots, stems and leaves were determined using a 4100-MPICP-OES (Agilent, Santa Clara, CA, USA) [22].

### Total RNA extraction and library construction

Total RNA from 18 samples was extracted with TRIzol reagent (Invitrogen). The quality and amount of the RNA was determined using a Nano Drop 2000 (Thermo Fisher Scientific Inc., USA) and an Agilent 2100 bioanalyzer (Agilent Technologies Inc., USA). The total RNA was used to construct a cDNA library. First, the mRNA containing a poly-A tail was selected using Oligo (dT) magnetic beads. Then double-stranded cDNA was synthesized by random hexamer reverse transcription. The 5' end of the double-stranded cDNA was repaired with the A-Tail Mix and RNA Index Adapter. Next, the ligated product was amplified by PCR with two specific primers, and the PCR product was thermally denatured into a single strand. The single-strand DNA was cyclized using splint oligonucleotides and DNA ligase to obtain the final library. Finally, transcriptome sequencing of the cDNA library was carried out on the BGISEQ-500 platform (BGI-Shenzhen, China) [23, 24], and single end 50 base pair reads were generated. The original sequencing reads have been submitted to the SRA at NCBI (Accession number: PRJNA611672).

### qRT-PCR

Total RNA was extracted from seedling roots of different treatment groups using the RNA Simple Total RNA Kit (Tiangen, Shanghai, China), according to the manufacturer’s instructions. The extracted RNA was reverse transcribed into cDNA using FastKing cDNA (Tiangen, Shanghai, China). Specific PCR primers for 10 genes were designed using primer-blast in NCBI. Amplification of samples and standards was carried out using the QuantStudio5 Real-time PCR System (Thermo Scientific, Massachusetts, USA) using SuperReal PreMix Plus (SYBR Green) (Tiangen, Shanghai, China). Three biological replicates were included. Each reaction was carried out in a 20 μL reaction volume, consisting of 10 μL Superreal PreMix Plus, 6 μL ddH_2_O, 0.8 μL forward primer (10 μmol/L), 0.8 μL reverse primer (10 μmol/L), 0.4 μL ROX reference Dye and 2 μL template cDNA. The amplification procedure was as follows: 95°C pre-denaturation for 10 min, 40 cycles of 15 s at 95°C, 30 s at 60°C. The melting curve conditions were as follows: 15 s at 95°C, 1 min at 60°C, and 15 s at 95°C. The relative transcription levels of selected genes was calculated using the 2^−ΔΔCt^ method [25], and normalized to the expression levels of the 18s gene [26]. qRT-PCR primers are shown in Supplementary S1 Table.

### Data analysis

All the data were statistically analyzed using SPSS 19.0 software, and the results were expressed as mean ± standard deviation (SD) and plotted using Microsoft Excel 2016.

A series of quality control steps were performed on the raw reads obtained by sequencing. The following reads were removed: reads containing adaptor sequence, reads with an unknown base number is greater than 5% of the total sequence length, and reads with Q ≤ 15, reads where the base number accounts for more than 20% of the total read base number of the reads. Then the clean reads for each sample were aligned to the third version of the B73 maize reference genome (http://ftp.maizesequence.org/) using HISAT software [27]. Gene expression levels were determined using RSEM software [28]. DEGs were identified as described by Audic *et al*. [29]. DEGs were defined as those with fold change in expression in S or T compared with CK > 1 or < -1, p-value < 0.05 and false discovery rate ≤ 0.001. The functional annotations of the selected DEGs in the GO database were determined using WEGO software [30]. The metabolic pathways enriched in DEGs was determined using the Kyoto Encyclopedia of Genes and Genomes pathway database (https://www.genome.jp/kegg/kegg1.html).

## Results

### Physiological indexes of maize seedlings under salt stress with and without GB mitigation

**Fig 1:**
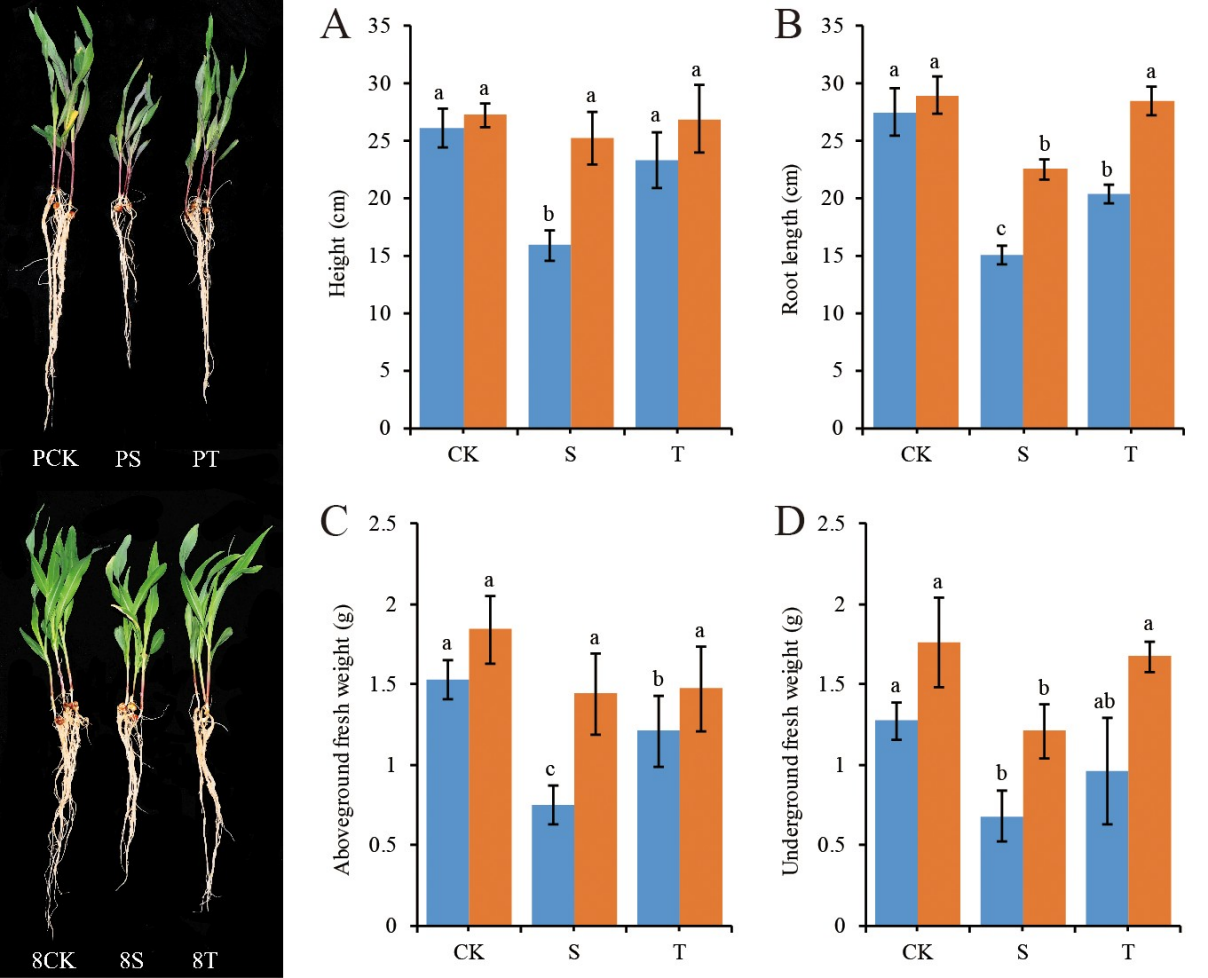
Changes in morphological indexes of P138 and 8723 seedlings under salt stress. The blue column represents P138 and the orange column represents 8723; different lowercase letters indicate a significant difference at the 5% level.

To study the physiological responses of two maize inbred lines to salt stress and the mitigation effect of exogenous GB, plant height, root length, aboveground fresh weight and underground fresh weight of these lines were measured. For both P138 and 8723, no significant difference in seedling growth was observed between seedlings grown under salt plus GB mitigation (T) treatment and those grown under normal conditions (CK), but P138 was obviously shorter under salt stress (S) treatment compared with CK, while 8723 showed little change, which indicated that GB had an obvious mitigation effect on the salt-sensitive maize genotype (Fig 1). The average values of plant height, root length, aboveground fresh weight and underground fresh weight for both varieties were highest under CK, followed by T and S, and the indexes of P138 changed more than those of 8723 (Fig 1A–1D).

### Changes in the ion contents of the two inbred lines under different treatments

**Fig 2:**
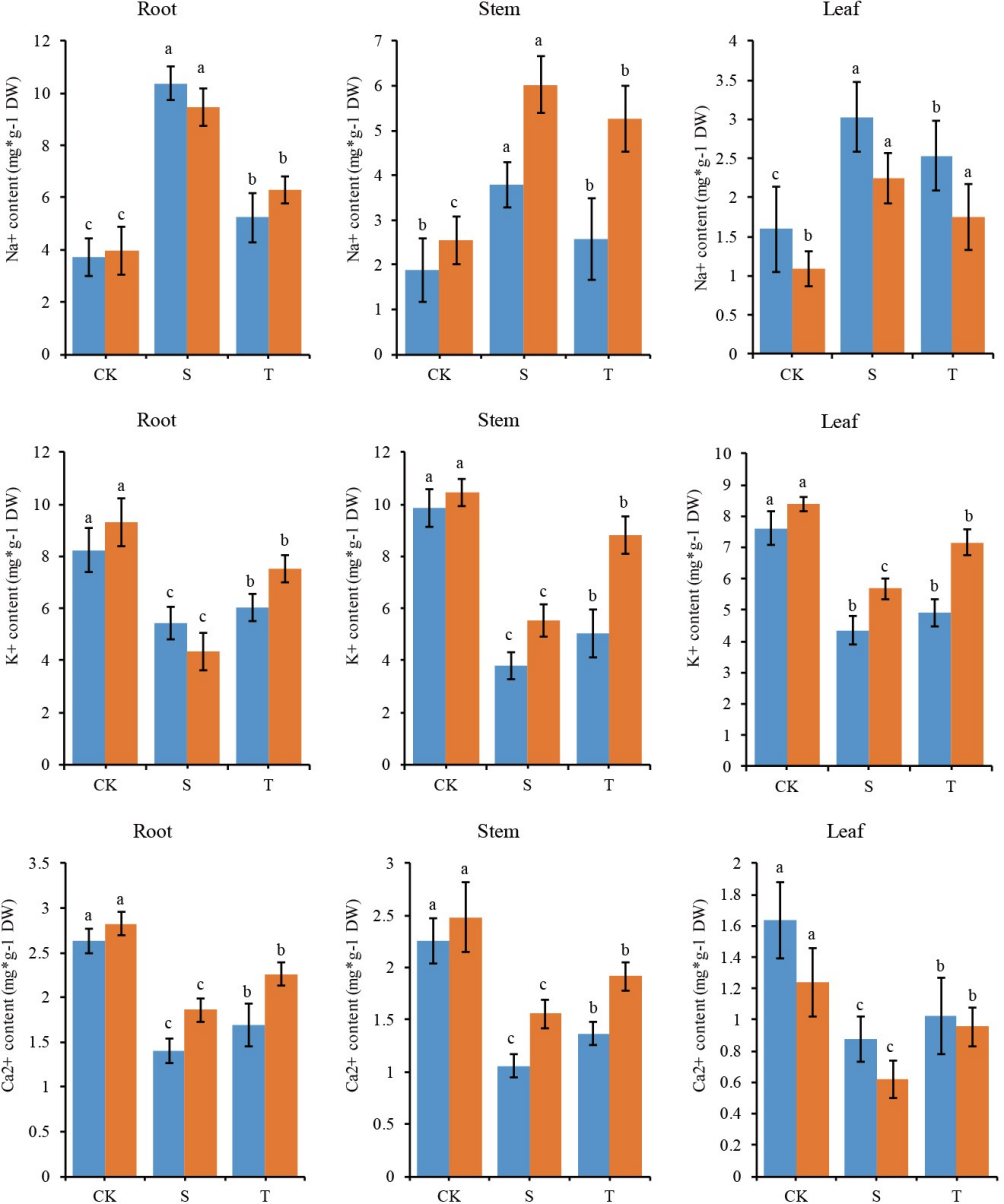
Changes in the ion contents in roots, stems and leaves of P138 and 8723 under salt stress. The blue column represents P138 and the orange column represents 8723.

To study the changes in the ion contents of the two inbred lines under salt stress with and without GB mitigation, the contents of Na^+^, K^+^ and Ca^2+^ in roots, stems and leaves were determined (Fig 2). P138 and 8723 had significantly higher Na^+^ contents in roots, stems and leaves under salt stress than under CK. When GB was added under salt stress, the content of Na^+^ decreased significantly, with a larger decrease observed in P138. P138 and 8723 had significantly lower contents of K^+^, Ca^2+^ in roots, stems and leaves under salt stress than under CK, and these contents increased significantly under the GB and salt stress treatment, with a larger change observed for P138. This shows that GB has an obvious mitigation effect on ion homeostasis under salt stress, and this effect is more obvious in the salt-sensitive inbred line P138.

### The change in the ratio of Na^+^, K^+^ and Ca^2+^ under different treatments

Table 1 shows that under the S and T treatments, the ratios of K^+^/Na^+^ and Ca^2+^/Na^+^ in all parts of maize seedlings were significantly lower compared with those under CK, but the ratios of K^+^/Na^+^ and Ca^2+^/Na^+^ in all parts of maize seedlings under the T treatment were significantly higher compared with those under the S treatment. Under the same treatment, the K^+^/Na^+^ ratios in different parts of the 8723 seedlings were higher than those in P138. Under the same treatment, the ratios of K^+^/Na^+^ and Ca^2+^/Na^+^ in the underground parts of the two varieties were lower than those in the aboveground parts, and the ratios of K^+^/Na^+^ and Ca^2+^/Na^+^ in the leaves were higher than those in the stems.

**Table 1:** Changes in the K^+^/Na^+^ and Ca^2+^/Na^+^ ratios of two maize inbred lines under different treatments at seedling stage.

		K^+^/Na^+^		Ca^2+^/Na^+^	
Part of seeding	Treatment	P138	8723	P138	8723
**Root**	**CK**	2.20a	2.35a	0.71a	0.71a
	**S**	0.52c	0.49c	0.14c	0.2b
	**T**	1.15b	1.19b	0.32b	0.36c
**Stem**	**CK**	5.25a	4.11a	1.2a	0.98a
	**S**	1.00c	1.18c	0.28c	0.26c
	**T**	1.95b	2.14b	0.53b	0.36b
**Leaf**	**CK**	4.76a	7.69a	1.02a	1.14a
	**S**	1.44c	2.52c	0.29c	0.27c
	**T**	1.94b	4.09b	0.4b	0.54b

Different lowercase letters indicate a significant difference at the 5% level.

### Transcriptome sequencing and sequence alignment

To ensure the reliability and comprehensiveness of the sequencing data, we measured the concentration and quality of total RNA extracted from each processed root sample, and found eighteen samples had good RNA integrity and were of high purity, and reached the standard required for RNA-sequencing (RNA-seq) (S2 Table). The BGISEQ-500 sequencing platform was used for RNA-seq, and an average of 124,096,423 original reads were generated per sample. Quality control was carried out to remove low-quality reads. The average number of clean reads was 110,263,267, accounting for 88.96% of the original reads (S3 Table). The screened clean reads were compared with the third version of the B73 maize reference genome. The average alignment rate for each sample was 75.84%, and most reads from each of the 18 samples were specifically aligned to the maize reference genome (only a small number of sequences aligned to multiple sites); in addition, the alignment rate between samples was uniform, indicating that the data were comparable between samples (S4 Table). The expression level of genes was determined using RSEM software. The average total number of expressed genes across the 18 samples was 35,493, of which most were known genes. The ratio of known genes to new genes is shown in Table 2.

**Table 2:** Gene expression in P138 and 8723 under different treatments.

Sample	Total Gene Number	Known Gene Number	Novel Gene Number	Known Gene Rate (%)	Novel Gene Rate (%)
**P138-CK1**	35752	30727	5025	85.94	14.06
**P138-CK2**	35779	30815	4964	86.13	13.87
**P138-CK3**	36171	31109	5062	86.01	13.99
**P138-S1**	35159	30415	4744	86.51	13.49
**P138-S2**	35929	30923	5006	86.07	13.93
**P138-S3**	35709	30739	4970	86.08	13.92
**P138-T1**	35380	30541	4839	86.32	13.68
**P138-T2**	35736	30820	4916	86.24	13.76
**P138-T3**	35241	30525	4716	86.62	13.38
**8723-CK1**	35791	30800	4991	86.06	13.94
**8723-CK2**	35809	30763	5046	85.91	14.09
**8723-CK3**	34513	30251	4262	87.65	12.35
**8723-S1**	36396	31265	5131	85.90	14.10
**8723-S2**	35523	30601	4922	86.14	13.86
**8723-S3**	34908	30232	4676	86.60	13.40
**8723-T1**	34191	30011	4180	87.77	12.23
**8723-T2**	35431	30548	4883	86.22	13.78
**8723-T3**	35443	30608	4835	86.36	13.64

### Venn diagram analysis of DEGs in P138 and 8723 under different treatments

**Fig 3:**
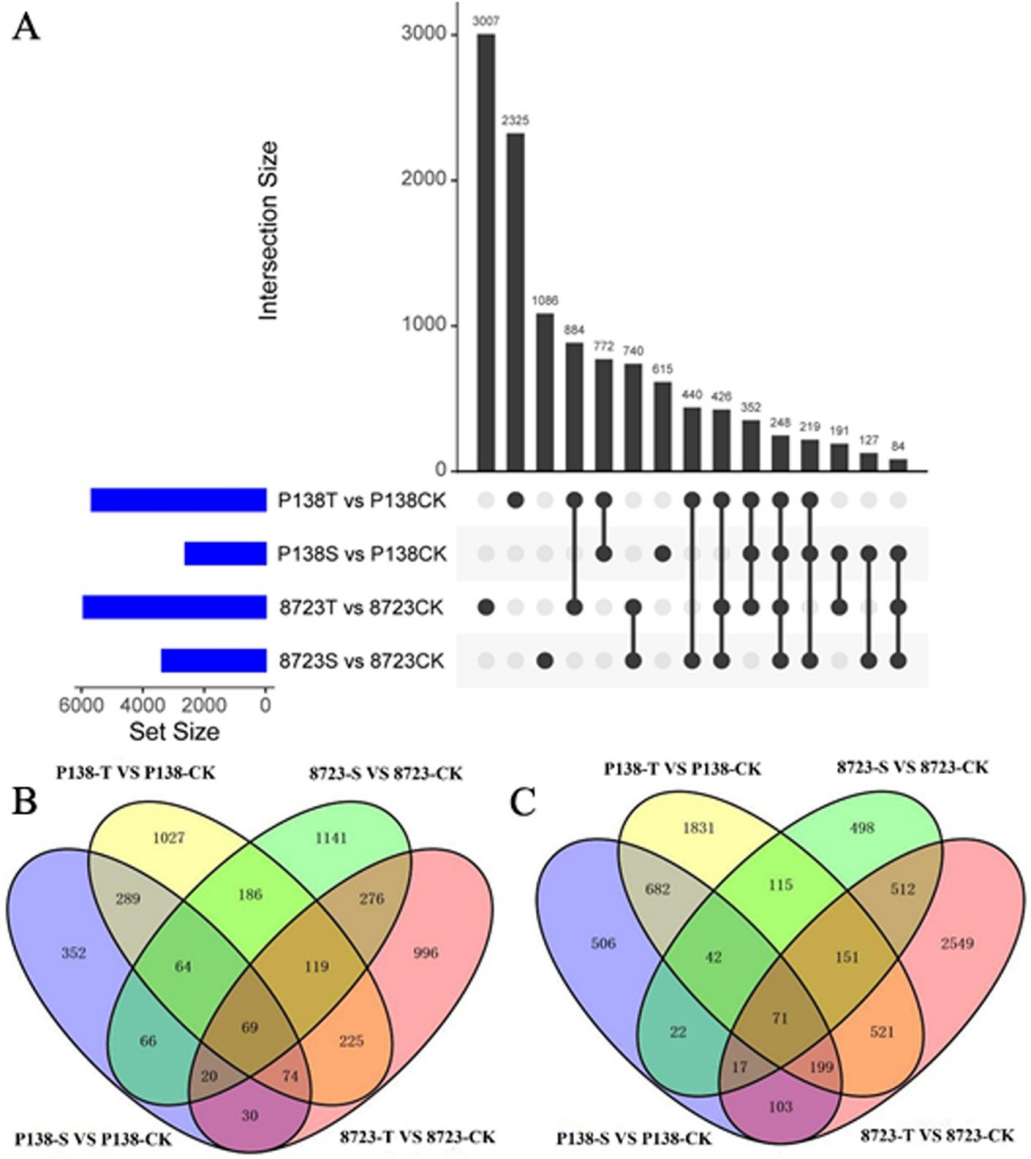
Venn diagram analysis of DEGs in P138 and 8723 under different treatments. (A) All DEGs for the three treatments in P138 and 8723. The bar charts indicate the number of DEGs under salt stress with and without the addition exogenous GB compared with CK; column charts indicate the number of DEGs under single or multiple processes. (B) Up-regulated DEGs identified in P138 and 8723 under both treatments (S and T); down-regulated DEGs identified in P138 and 8723 under both treatments (S and T).

To comprehensively study the response of salt-tolerant maize to salt stress and the mitigative effect of exogenous GB on salt stress-induced damage, we identified DEGs (|Fold-change in expression| >1 and p-value <0.05) under these conditions using FPKM as a measure of gene expression. A total of 7550 and 8561 DEGs were identified in P138 and 8723 under both treatments (S and T), respectively (Fig 3A). The Venn diagram reflects the distribution of up- and down-regulated DEGs in the two inbred lines under salt stress with exogenous GB treatment compared with the CK treatment (Fig 3B and 3C). Under salt stress, 2607 DEGs (965 up-regulated and 1642 down-regulated) were identified in P138 (S5 and S6 Tables), and 3369 DEGs (1941 up-regulated and 1428 down-regulated) were identified in 8723 compared with CK (S7 and S8 Tables). Under the T treatment, 5665 DEGs (2053 up-regulated and 3612 down-regulated) were identified in P138 (S9 and S10 Tables), and 5932 DEGs (1809 up-regulated and 4123 down-regulated) were identified in 8723 compared with CK (S11 and S12 Tables). And a total of 496 up-regulated DEGs and 994 down-regulated were identified in both the P138S vs P138CK and P138T vs P138CK comparisons, and 484 up-regulated and 751 down-regulated DEGs were identified in both the 8723S vs 8723CK and 8723T vs 8723CK comparisons. A total of 69 up-regulated DEGs (S13 Table) and 71 down-regulated DEGs (S14 Table) were identified in both inbred lines under both treatments (S and T). These shared DEGs may play an important role in salt tolerance and exogenous GB mitigation in maize, and reflect similar responses of the two materials to salt stress.

### Gene ontology functional classification of DEGs in P138 and 8723 roots under different treatments

**Fig 4:**
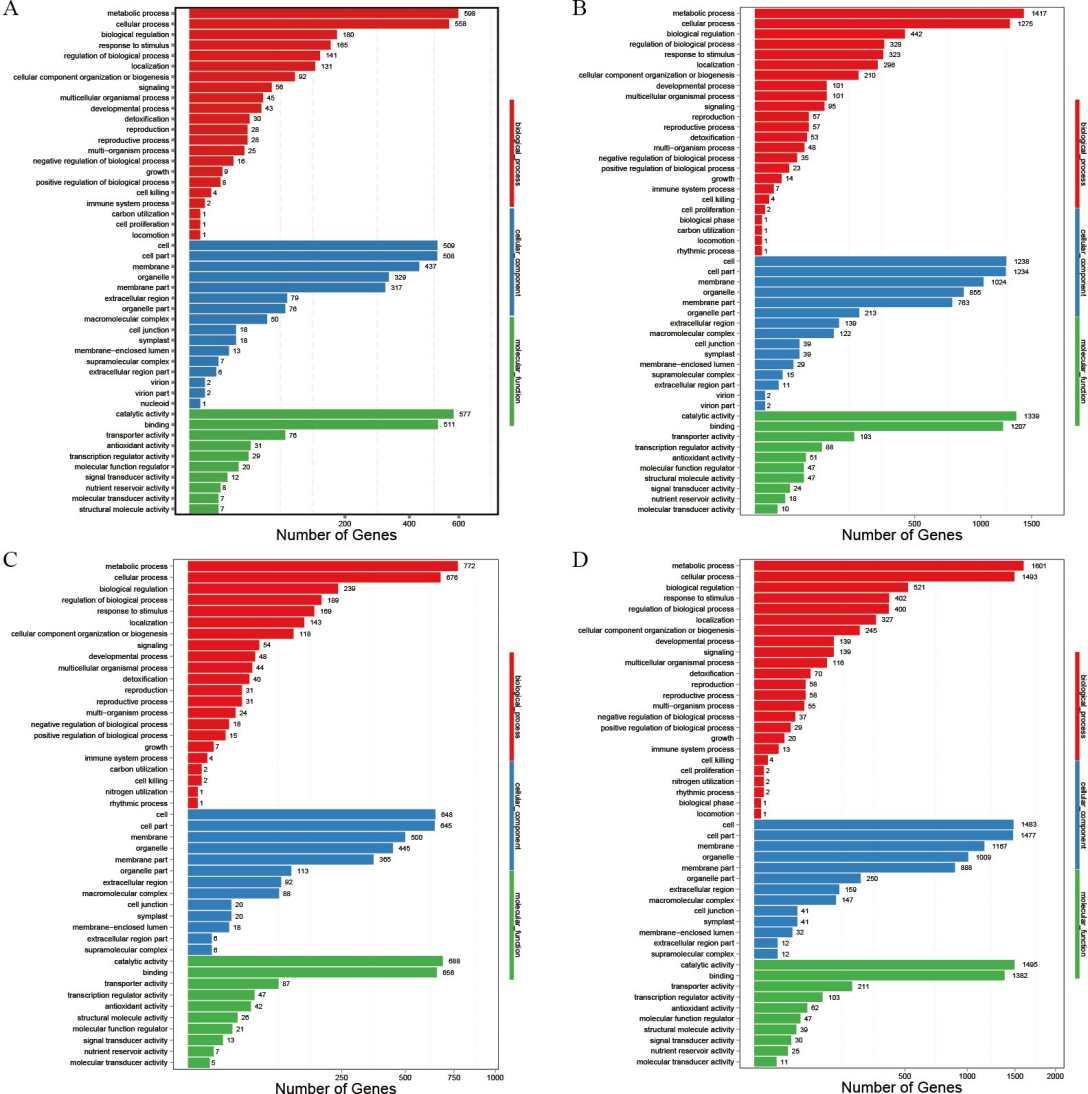
GO annotations of DEGs in P138 and 8723 identified under different treatments. A: P138S *vs* P138CK, B: P138T *vs* P138CK, C: 8723S *vs* 8723CK, D: 8723T *vs* 8723 CK.

All the DEGs were analyzed using gene ontology (GO) functional annotations, which are divided into biological process, cellular component, and molecular function categories (Fig 4). The DEGs in P138 under salt stress were mainly annotated to the biological process terms cellular process, metabolic process and response to stimulus. The main cell component terms were cell, cell partial, membrane, membrane partial and organelle. The main molecular function terms were catalytic activity and binding. Under salt stress, the main GO terms for 8723 were the same as those for P138, except that 8723 had more DEGs annotated to the same GO term. This indicates that 8723 may prevent salt stress damage by altering the expression of more genes. In addition, more DEGs annotated to each GO term were identified in the T vs CK comparison than in the S vs CK comparison. This also indicates that GB may alleviate salt stress damage in maize by altering the expression of more genes.

### Pathway enrichment analysis of P138 and 8723 under different treatments

**Fig 5:**
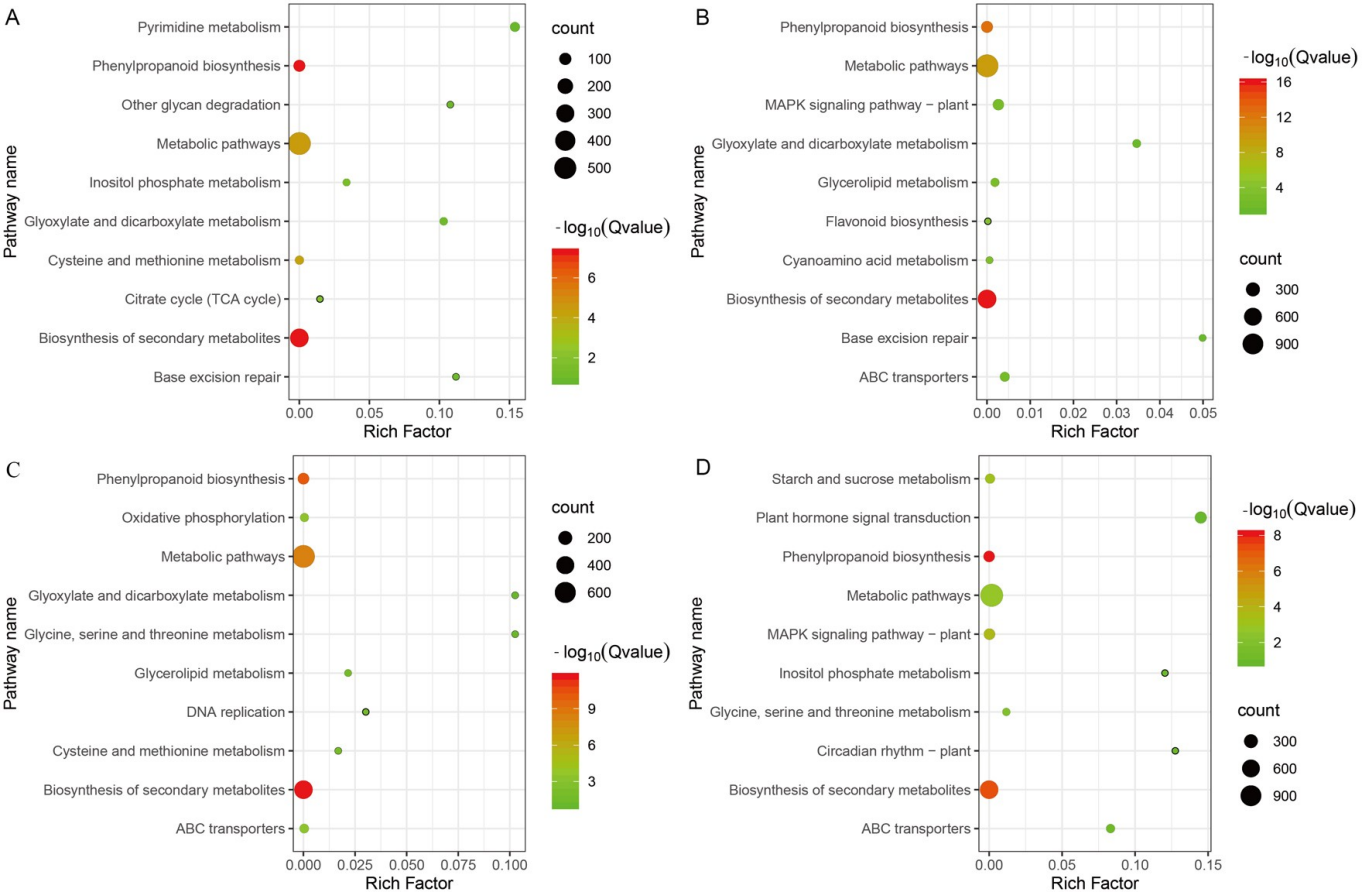
Pathway enrichment analysis of P138 and 8723 under different treatments. A: P138S *vs* P138CK, B: P138T *vs* P138CK, C: 8723S *vs* 8723CK, D: 8723T *vs* 8723 CK.

We performed pathway enrichment analysis of all DEGs in the two inbred lines, and selected the top 10 metabolic pathways from each comparison (Fig 5). DEGs identified from the P138S *vs* P138CK and 8723S *vs* 8723CK comparisons were enriched in the metabolic pathways, biosynthesis of secondary metabolites, and phenylpropanoid biosynthesis. Under T treatment, DEGs were enriched in metabolic pathways, biosynthesis of secondary metabolites, and plant hormone signal transduction in 8723, and in metabolic pathways, biosynthesis of secondary metabolites, and phenylpropanoid biosynthesis in P138. There were a larger number DEGs from the S *vs* CK comparison that were annotated to the same metabolic pathway in the two inbred lines compared with those in the T *vs* CK comparison. These DEGs may be the key for the alleviation of salt stress-induced damage by exogenous GB. The DEGs from the S *vs* CK comparison unique to 8723 included ABC transporters, and genes involved in oxidative phosphorylation, glycerolipid metabolism, glycine, serine and threonine metabolism, and DNA replication. The differential regulation of these genes specifically in 8723 may be one of the reasons for the higher salt tolerance of 8723.

Expression analysis combined with physiological analysis revealed that GB had a more obvious mitigation effect on salt-sensitive P138 under salt stress. Therefore, we focused on the changes in P138 pathways under exogenous GB treatment. Under T treatment, the DEGs unique to P138 under salt stress were annotated to MAPK signaling pathway-plant, ABC transporters, glycerolipid metabolism, cyanoamino acid metabolism, flavonoid biosynthesis. Therefore, we speculate that these unique pathways enhance the tolerance of P138 to salt stress.

### Transcription factor family analysis

**Fig 6:**
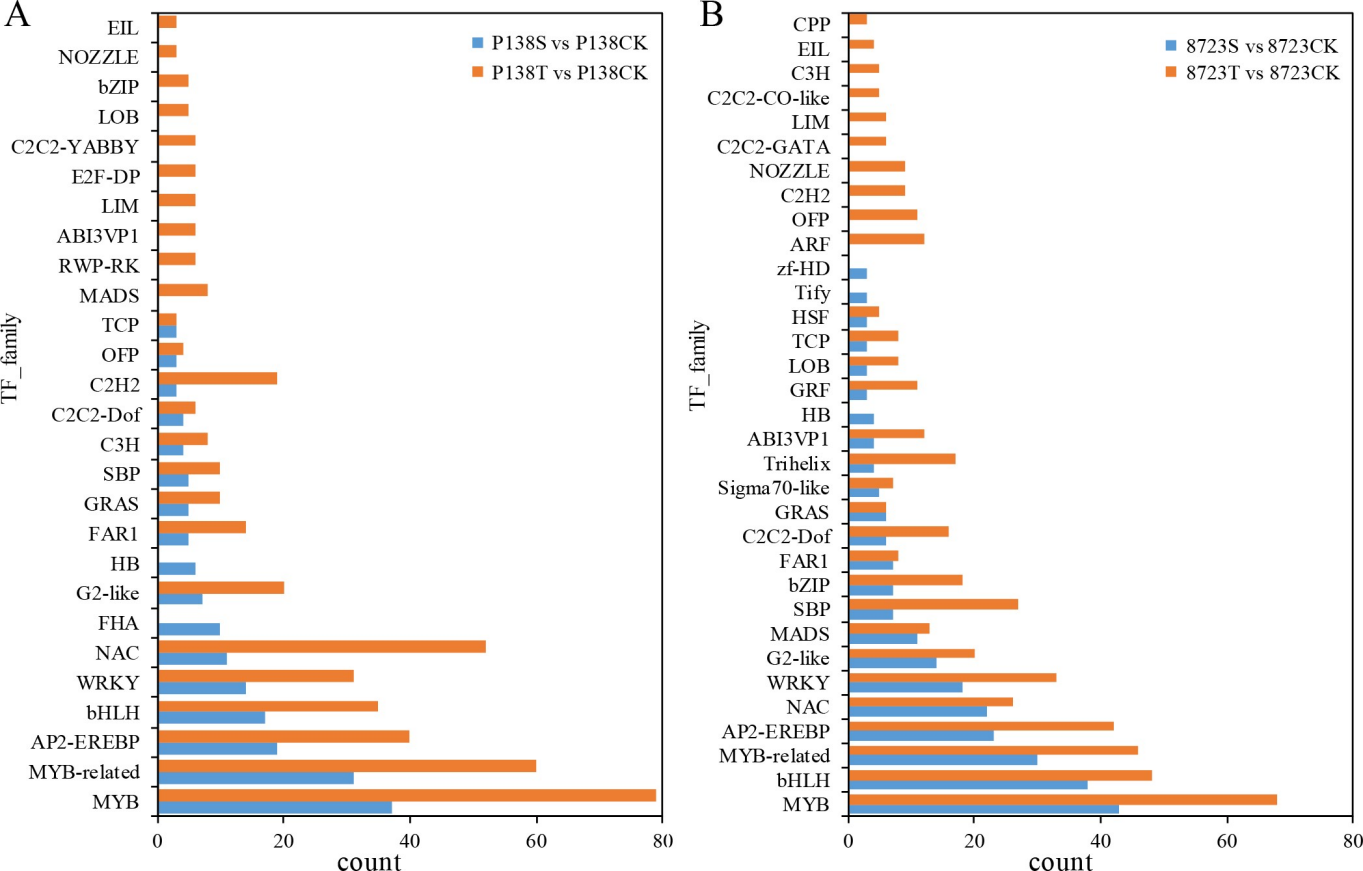
Analysis of transcription factors differentially expressed in P138 and 8723 under different treatments.

TFs are the key to the regulation of gene expression in plants under environmental stress. RNA-seq results showed that many TFs in 8723 and P138 were differentially regulated under the S and T treatments. Here we only show the TF families with more than two DEGs (Fig 6). We found that compared with CK, TFs in 27 families were differentially regulated in P138 under salt stress (Fig 6A). The three TF families with the most DEGs were MYB, MYB-related and AP2-EREBP. In 8723 TFs in 32 families were differentially regulated under salt stress, with the most belonging to the MYB, bHLH and MYB-related TF families (Fig 6B). In the T *vs* CK comparison, TFs in 37 families were differentially expressed in P138, most of which belonged to the MYB, MYB-related and NAC families. In 8723 members of 44 TF families were differentially expressed, and most were members of the MYB, bHLH and MYB-related families. For both lines, more TF DEGs were identified in the T *vs* CK comparison than in the S *vs* CK comparison, and more genes belonged to the same TF family, indicating that changes in the expression of these TF genes may be the key to GB alleviation of salt stress damage in maize.

### Validation of DEGs by qRT-PCR analysis

**Fig 7:**
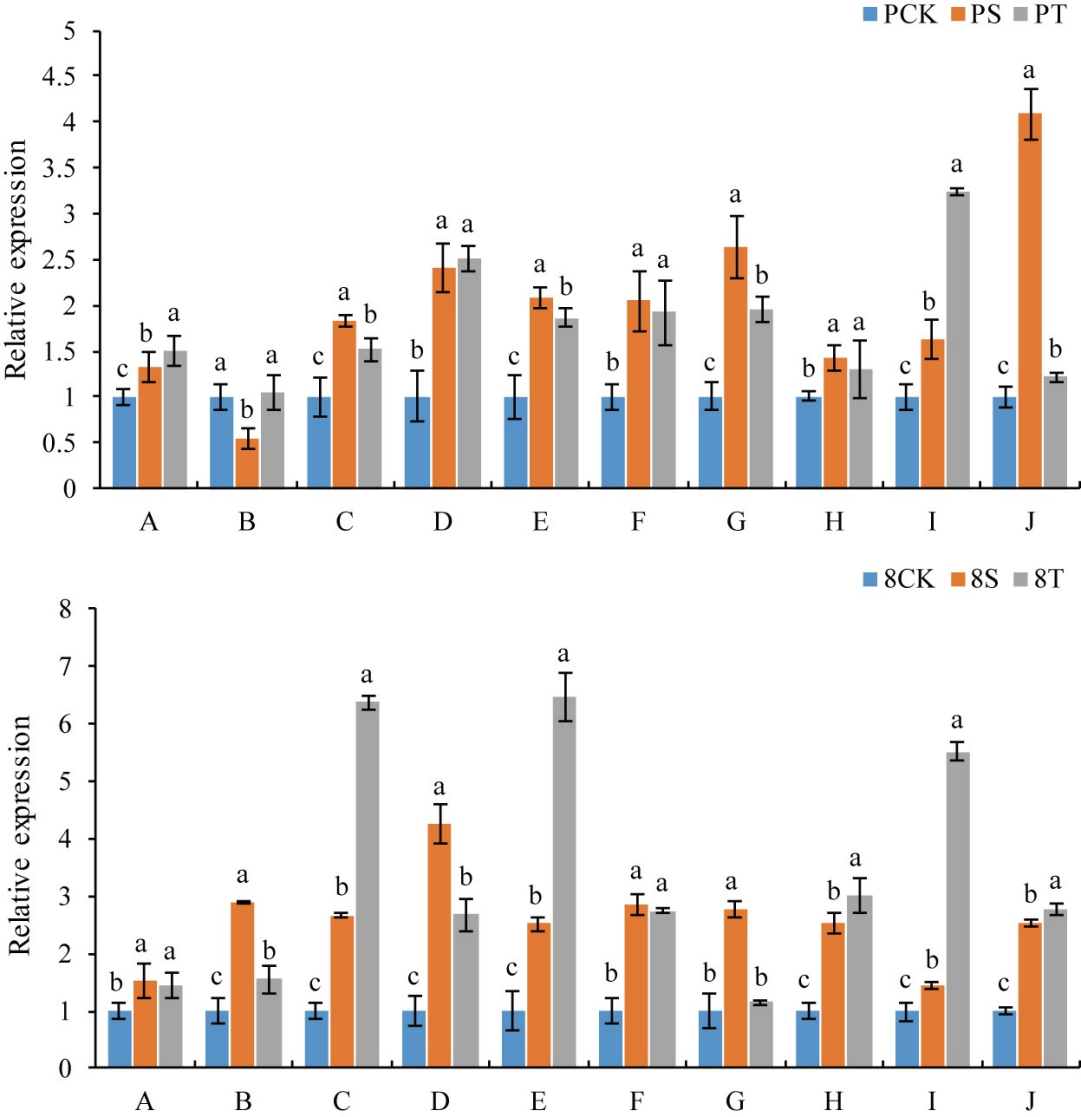
The relative abundance of selected genes was verified by qRT-PCR. A: Seven-transmembrane-domain protein 1, B: DNA binding protein, C: putative O-Glycosyl hydrolase superfamily protein, D: putative alpha-amylase family protein, E: Sulfate transporter 1.2, F: POU domain, class 3, transcription factor 3, G: Glycine-rich cell wall structural protein 2-like, H: Protein P21, I: Probable carboxylesterase 15, J: Potassium transporter 21-like. Different lowercase letters indicate a significant difference at the 5% level.

To evaluate the reliability of gene expression profiles in response to salt stress with and without the addition exogenous GB to alleviate salt stress damage, 10 genes up-regulated in P138 and 8723 under the S and T treatments were verified by qRT-PCR (Fig 7 and S15 Table). We found that the expression levels of these 10 genes in the two inbred lines were basically consistent with the results of transcriptome sequencing, indicating that our RNA-seq data are reliable.

## Discuss

### Physiological analysis of salt tolerance

Salt stress has a very significant effect on plant development. Excessive salt in soil causes osmotic stress and inhibits plant growth, which is usually manifested as by a decrease in plant biomass and the rate of leaf area expansion [31, 32]. Maize is more severely affected by salt stress during the process of morphogenesis at the seedling stage. We found that the growth of maize seedlings was inhibited, and that plant height, root length, seedling fresh weight and root fresh weight were significantly decreased under salt stress, which is consistent with the results of previous studies [33]. Under salt treatment, all morphological parameters of P138 were significantly different compared with those of the control, but only the root length and underground fresh weight of 8723 were significantly different compared with the control, which indicates that the degree of inhibition of maize growth by salt stress is related to the characteristics of the material itself.

Salt stress can cause osmotic stress and ion stress damage in plants [34]. Maintaining K^+^ and Na^+^ homeostasis under salt stress is very important for plant tolerance to salt stress [35]. Previous studies have shown that Na^+^ and K^+^ have similar a ion radius and a similar hydration energy, and they compete with each other for the same binding sites on transporters [36]. Under salt stress, the increase of Na^+^ content in plants significantly inhibits the absorption of K^+^ [37, 38]. We found that the Na^+^ content of the two maize inbred lines increased under salt stress, while the content of K^+^ decreased, indicating that when the content of Na^+^ in the external environment is high, the absorption of K^+^ is significantly inhibited. This is due to the increase of plasma membrane permeability caused by high concentrations of Na^+^, which leads to a decrease in K^+^ absorption. Ca^2+^ is one of the most important second messengers in plant cell signal transduction pathways. In this study, we found that the content of Ca^2+^ in the roots and stems of 8723 was significantly higher than that in the roots and stems of P138; the higher content of Ca^2+^ in inbred line 8723 could promote the absorption of K^+^ in the stem and reduce the damage caused by salt stress. In addition, we found that the content of Na^+^ in the roots and stems of P138 was lower than that in the roots and stems of 8723 under salt stress, in contrast to leaves. Under T treatment, the content of Na^+^ in roots, stems and leaves of the two inbred lines decreased compared with that under salt stress, but the decrease in 8723 was less than that in P138, indicating that roots and stems of 8723 could accumulate more Na^+^ under salt stress. This would prevent it from being transported to leaves, keeping the Na^+^ content in leaves low, and reducing Na^+^ damage to aboveground parts. However, P138 could not store more Na^+^ in the root and stem, which led to a higher flow of Na^+^ from the roots to the leaves, resulting in an increase in the Na^+^ content in the leaf and injury. The addition of an appropriate concentration of exogenous GB effectively regulated ion homeostasis in the two inbred lines, which was consistent with the results of previous studies [39, 40]. We also found that the decrease of K^+^ content in 8723 roots was higher than that in P138 roots under salt stress, but the opposite trend was observed in stems and leaves, indicating that inbred line 8723 had a stronger ability to selectively transport K^+^ than P138. Through the selective transport of K^+^, the transport of Na^+^ to the stems and leaves was inhibited, thus keeping the K^+^/Na^+^ ratio at a relatively stable level and preventing salt damage; this finding is similar to the findings of previous studies [41, 42].

K^+^/Na^+^ and Ca^2+^/Na^+^ are important indexes reflecting ion balance and injury in plants [43, 44]. We found that the contents of K^+^/Na^+^ and Ca^2+^/Na^+^ in all parts of seedlings of the two inbred lines decreased significantly under salt stress, and increased significantly after the addition of exogenous GB. The results showed that the Ca^2+^/Na^+^ ratio of the underground parts was lower than that of the aboveground parts of the two maize inbred lines under salt stress, but the Ca^2+^/Na^+^ ratio of 8723 seedlings was higher than that of P138. This finding is similar to that of of Zhao *et al*. [45], who proposed that salt-tolerant plants maintain intracellular ion balance under salt stress by maintaining higher K^+^/Na^+^ and Ca^2+^/Na^+^ ratios, thereby reducing the damage caused by salt stress.

### Transcriptome analysis of salt tolerance

Transcriptome profiling has been widely and successfully used to analyze salt stress response mechanisms of plants. It is an effective strategy to identify common sets of genes that are differentially expressed between stress-tolerant and sensitive genotypes with different genetic backgrounds [15, 46, 47]. Comparing the difference in transcriptional levels between tolerant and sensitive genotypes under stress conditions, the genes related to stress tolerance can be isolated [48]. For example, Peng *et al*. [15] used a comparative transcriptome method to identify DEGs in leaves of salt-tolerant and salt-sensitive cotton genotypes, and found that 819 TF genes were differentially expressed in the two genotypes and that 129 genes were specifically expressed in the salt-tolerant genotypes 24 hours after salt stress. In this study, we found 219 up-regulated and 153 down-regulated DEGs in salt-sensitive P138 and salt-tolerant 8723 under NaCl treatment. Under T treatment, there were 487 up-regulated and 942 down-regulated DEGs in P138 and 8723, and 69 up-regulated and 71 down-regulated DEGs were identified in P138 and 8723 under both the S and T treatments. The fact that DEGs with the same profiles were found in different materials under S or T treatment shows that different materials share common responses to salt stress.

#### Ion transport

Salt stress disrupts the ion balance in plants, affecting physiological and biochemical metabolism [37, 38]. It is critical to maintain a high K^+^/Na^+^ ratio in plant tissues to improve salt tolerance [49]. We found that a metal ion binding protein gene (LOC100281997) was down-regulated in P138 under salt stress, but up-regulated in 8723. The protein encoded by this gene mainly regulates the transport of charged metal ions inside and outside and between cells, indicating that this gene may enhance 8723 salt tolerance by affecting metal ion balance. High salt conditions cause competition between Na^+^ and K^+^, resulting in K^+^ deficiency, but plants may increase K^+^ uptake through KUP family proteins and thus improve salt tolerance [50, 51]. In this study, we found that a KUP family potassium transporter gene (Potassium transporter 21-like, LOC103643618) was up-regulated in two inbred lines under salt stress, and the expression of this gene increased after the addition of GB, indicating that Potassium transporter 21-like could improve salt tolerance by regulating plant potassium absorption and that the addition of exogenous GB may enhance maize salt stress tolerance by promoting the expression of this transporter. In addition, a phosphate transporter protein gene (LOC100502494) was also up-regulated in the two inbred lines under salt stress, and its expression increased after the addition of GB; therefore, so we speculate that this gene has a positive effect on plant salt tolerance.

#### Secondary metabolism

Plants can accumulate a large number of secondary metabolites during growth and development; these metabolites not only play a role in regulating normal growth and development, but also in regulating the adaptability of plants to stressful environments [52, 53]. Cytochrome P450 is an important monooxygenase involved in xenobiotic metabolism and steroid biosynthesis [54]. Previous studies have found that up-regulation of cytochrome P450 expression under salt stress can enhance the salt tolerance of *Arabidopsis thaliana* [55]. In this study, we found that the expression of a cytochrome P450 family 2 subtype gene (Ent-cassadiene C2-murhydroxylase-like, LOC103626064) was significantly up-regulated in the two inbred lines under T treatment. Because this gene is involved in several oxidative metabolic processes, we suggest that it may enhance the salt tolerance of maize when its expression induced by GB. Previous studies have found that the accumulation of dihydroxy B-ring flavonoids can improve the salt tolerance of soybean (*Glycine max*) [56]. In this study, we found that the expression of one cyanidin 3-O-rutinoside 5-O-glucosyltransferase gene (LOC103630119) increased significantly in the two inbred lines under T treatment. Because this gene is involved in flavonoid biosynthetic process, we speculate that this gene may improve the salt tolerance of plants by regulating the metabolism of polysaccharides.

#### Reactive oxygen scavenging mechanism

Under salt stress, excessive accumulation of ROS damages cell membranes and related biological macromolecules. Using antioxidant enzyme systems to minimize oxidative damage is one of the strategies employed by plants to adapt to stress [57, 58]. Peroxidase is an antioxidant enzyme that when up-regulated under salt stress can alleviate the toxic effect of excessive ROS accumulation in cells [59]. We identified two peroxidase-related genes up-regulated in P138 under salt stress, peroxidase 2 (LOC103633480) and L-ascorbate peroxidase 1 (LOC103634427), one peroxidase gene, peroxidase 67 (LOC100285577), up-regulated in both inbred lines. Cationic peroxidase-1 (LOC103633557) was down-regulated in P138 but up-regulated in 8723 under salt stress. In addition, we also found that the expression of APx1-Cytosolic ascorbate peroxidase (LOC100283822) and peroxidase 2-like (LOC103633483) was up-regulated in 8723 under salt stress, and in both inbred lines under T treatment. The increase in the expression of these genes under salt stress may enhance the activity of the encoded enzymes, reduce the levels of ROS produced by salt stress, and protect plants from damage. Glutathione S-transferases (GSTs) can promote cell survival under oxidative stress by protecting cells from oxygen toxicity and inhibiting apoptosis. It has been confirmed that the up-regulated expression of a GST gene improves salt tolerance in transgenic *A*. *thaliana* plants [60]. We found that GST 9 (LOC542629) was up-regulated in 8723 under salt stress and may contribute to resistance to oxidative toxicity induced by salt stress. These results indicate that there are potential differences between the two varieties in the protective mechanisms used to protect against salt stress-induced oxidative damage.

#### Signal transduction

Adapting to stress by regulating complex signaling networks is another coping strategy used by plants [61]. Pathogenesis-related proteins are involved in plant responses to various pathogens and environmental stresses, and play an important role in plant defense systems [62, 63]. We found that pathogenesis-related protein 1 (LOC103634525), which is involved in the abscisic acid-activated signaling pathway, was up-regulated in the two inbred lines under salt stress. Ubiquitin mainly regulates the post-translational modification of eukaryotic proteins and participates in plant responses to abiotic stress [64]. We found that ubiquitin carrier protein 7 (LOC103635029) was up-regulated in both inbred lines under salt stress. At present, many protein kinases have been found to be involved in plant responses to stress, such as Ca^2+^ dependent protein kinase 3 *CPK3* [65], *OsCPK12* [66], and the CBL-interacting protein kinase *TaCIPK29* [67]. We found that three protein kinase-related genes [putative calcium-dependent protein kinase family protein (LOC100383301), CBL-interacting protein kinase (LOC100141385), and CBL-interacting protein kinase 14-like (LOC103640734)] were up-regulated in both inbred lines under T treatment; these genes are annotated as being involved abscisic acid-activated signaling pathways, signal transduction, and steroid hormone mediated signaling pathways. This indicates that GB may enhance the stress tolerance of maize by inducing the expression of many protein kinase genes.

The MAPK (mitogen-activated protein kinases) cascade plays an important role in intracellular pathogen immunity and abiotic stress signal transduction. For example, the MAPK cascade plays a key role in enzyme activation and inactivation through phosphorylation / dephosphorylation, which makes it possible for rapid and specific signal transduction and amplification of external stimuli [68]. Protein phosphatase 2C gene can increase the salt tolerance of *Arabidopsis thaliana* through positive regulation in abscisic acid (ABA) pathway [69]. In this study, we identified a putative protein phosphatase 2C family protein gene (LOC 100382524) that was up-regulated in the ABA pathway of the MAPK cascade under the T treatment in P138, but was not identified under the salt stress. we suggest that the gene may be induced by exogenous GB, which enhances the salt tolerance of P138. In addition, previous studies have found that an *Arabidopsis* Raf-like MAPKKK gene *Raf43* plays an important role in biotic and abiotic stress [70]. We identified that a mitogen-activated protein kinase kinase kinase 18 gene (LOC 103634498) in the ABA pathway was up-regulated under both treatments (S and T) in P138, but the expression level was higher under the T treatment. Therefore, we speculate that exogenous GB enhances the salt tolerance of maize by enhancing the expression of this gene, and the gene may be a positive regulatory gene in the ABA pathway response to salt stress.

#### Response of TF family members to salt stress

TFs that control the expression of stress response genes are very important in regulating plant responses to a range of abiotic and biological stressors [71]. Under both treatments (S and T), most TFs differentially expressed in the two maize inbred lines were members of the MYB, MYB-related, AP2-EREBP, NAC, and WRKY TF families. Many genes encoding MYB TFs are up- or down-regulated in response to salt stress [72]. In this study, more MYB family TFs were differentially expressed in 8723 under salt stress than in P138, while after T treatment, more MYB TFs were differentially expressed in P138 than in 8723. This indicates that GB may improve the salt tolerance of P138 by inducing the expression of MYB family TFs.

NAC TFs play an important role in plant response to salt stress, and it has been found that overexpression of NAC genes can significantly improve the salt tolerance of plants [73, 74]. In our study, we found that more NAC TFs were differentially expressed in P138 under the T treatment, which may indicate that GB enhances the salt tolerance of P138 by inducing NAC TF expression. For example, uncharacterized LOC100502408, NAC domain-containing protein 43 (LOC103628975), NAC transcription factor (100857067), uncharacterized LOC100279912, and putative NAC domain-containing protein 61 (LOC103630400) were up-regulated in 8723 under salt stress, but up-regulation was only observed in P138 after T treatment.

bZIPs are one of the largest TFs families in plants. Studies have found that some bZIP-TFs are involved in salt stress signal transduction. For example, ABRE binding factors can respond to salt stress at both the transcriptional and post-transcriptional levels [71]. We found that under salt stress, 7 bZIP-TFs were differentially expressed in 8723 under salt stress, with the number increasing to 18 under T treatment, while no bZIP-TF differential expression under salt stress was observed in P138. Five differentially regulated TFs (4 down-regulated, 1 up-regulated) were identified under the T treatment; and one of the up-regulated TFs was an ABRE binding protein (LOC100502540), which is mainly involved in plant stress response. This suggests that these TFs may be induced by GB, thus enhancing salt tolerance.

WRKY TFs belong to a very large family, members of which are mainly involved in plant growth and development and response to abiotic stress [75, 76]. We found that two WRKY TFs [WRKY 64, (LOC100281232), probable WRKY transcription factor 72, (103639796)] were up-regulated in the two inbred lines under salt stress, and the expression of these TFs increased after T treatment, indicating that the two WRKY TFs may regulate the tolerance of maize to salt stress.

#### Model of the molecular mechanisms underlying salt tolerance and GB mitigation of salt stress-induced damage in maize

**Fig 8:**
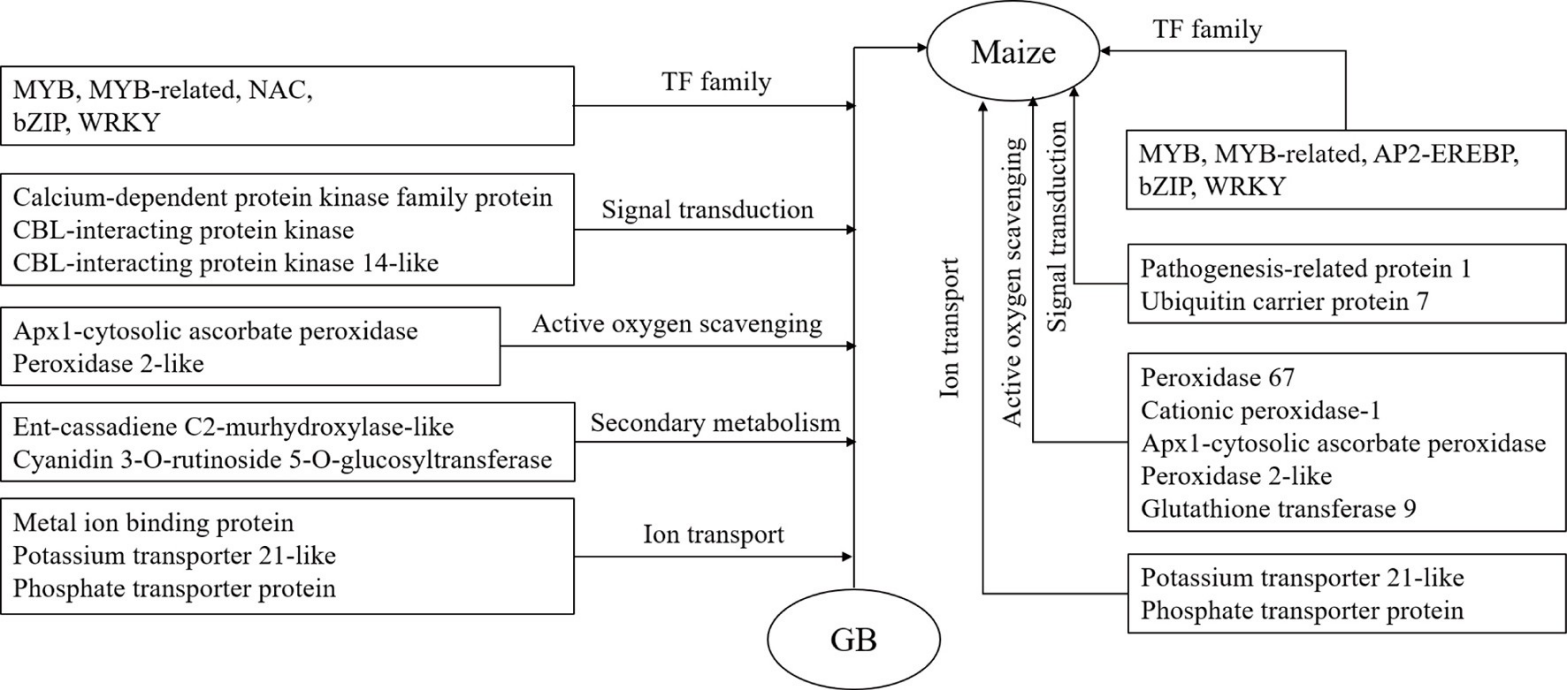
Molecular model of the mechanisms underlying salt tolerance and GB regulation of salt tolerance in maize seedling roots.

Based on comparative transcriptome analysis and investigation of the physiological differences of two inbred lines with different salt tolerances grown under salt stress with and without GB treatment, we established the following molecular model of the mechanisms underlying salt tolerance and GB regulation of salt tolerance in maize (Fig 8). In 8723 the response to salt stress is mainly achieved through stabilizing ion homeostasis, strong signal transduction activation, increasing reactive oxygen scavenging, and increasing the expression of TFs involved in the regulation of stress-tolerance genes. In addition, GB alleviates salt stress in maize mainly by inducing gene expression changes to enhance the ion balance, secondary metabolic level, reactive oxygen scavenging mechanism, signal transduction activation and the expression of more TFs, so as to improve the ability of maize to tolerate salt stress.

## Conclusion

The differences in the morphology, physiological and biochemical indexes, and root transcription profiles of inbred line P138 and inbred line 8723 under salt stress with and without GB treatment were compared. We found that the growth of P138 was significantly inhibited and the in vivo ion balance was disrupted under salt stress, whereas 8723 could prevent salt injury by maintaining a high ratio of K^+^ to Na^+^. The addition of a suitable concentration of GB could effectively alleviate the damage caused by salt stress, and the mitigation effect of GB on salt-sensitive inbred line P138 was more obvious than that on 8723. In addition, 219 genes were up-regulated and 153 were down-regulated in both P138 and 8723 under salt stress, and 487 genes were up-regulated and 942 were down-regulated in both P138 and P1380 under T treatment. These genes regulate plant responses to salt stress mainly through their involvement in ion transport, signal transduction, secondary metabolism and reactive oxygen species scavenging. In addition, the TFs differentially regulated in response to salt stress and exogenous GB mitigation mainly belong to the MYB, MYB-related, AP2-EREBP, bHLH, and NAC families.

## Supporting information

S1 Table Primer sequences used for qRT-PCR analysis in this article. (XLSX)

S2 Table Quality control of the total RNA for RNA-Seq. (XLSX)

S3 Table Summary of sequencing data. (XLSX)

S4 Table Summary of genome mapping information. (XLSX)

S5 Table DEGs up-regulated in P138 seedling roots under S treatment. (XLSX)

S6 Table DEGs down-regulated in P138 seedling roots under S treatment. (XLSX)

S7 Table DEGs up-regulated in 8723 seedling roots under S treatment. (XLSX)

S8 Table DEGs down-regulated in 8723 seedling roots under S treatment. (XLSX)

S9 Table DEGs up-regulated in P138 seedling roots under T treatment. (XLSX)

S10 Table DEGs down-regulated in P138 seedling roots under T treatment. (XLSX)

S11 Table DEGs up-regulated in 8723 seedling roots under T treatment. (XLSX)

S12 Table DEGs down-regulated in 8723 seedling roots under T treatment. (XLSX)

S13 Table DEGs up-regulated in both inbred lines under S and T treatments. (XLSX)

S14 Table DEGs down-regulated in both inbred lines under S and T treatments. (XLSX)

S15 Table DEGs used for qRT-PCR analysis. (XLSX)
